# An overview of solid-state electron paramagnetic resonance spectroscopy for artificial fuel reactions

**DOI:** 10.1016/j.isci.2022.105360

**Published:** 2022-10-14

**Authors:** Jeremy A. Bau, Abdul-Hamid Emwas, Magnus Rueping

**Affiliations:** 1 KAUST Catalysis Center, Physical Sciences and Engineering Division, King Abdullah University of Science and Technology (KAUST), Thuwal 23955, Saudi Arabia; 2 KAUST Core Labs, KAUST, Thuwal 23955, Saudi Arabia

**Keywords:** Chemistry, Materials science, Materials chemistry

## Abstract

The use of spectroscopy to characterize electrocatalytic processes is vital to the understanding and continuing the development of new catalysts for clean energy transformations. Electron paramagnetic resonance spectroscopy (EPR), which allows for the study of unpaired electron spins, shows great fundamental promise for the study of electrocatalysts, but was previously hindered by design limitations. Recently, several groups have demonstrated that these limitations can be overcome, providing valuable understandings of electrocatalyst function that other techniques are less suitable for. In this review, we summarize these findings across a range of experimental approaches and systems and describe the importance of EPR to each of these studies. By providing outlines for how these studies were able to overcome experimental design challenges, we hope to provide insight into potentially interested users.

## Graphical abstract

**Figure:**
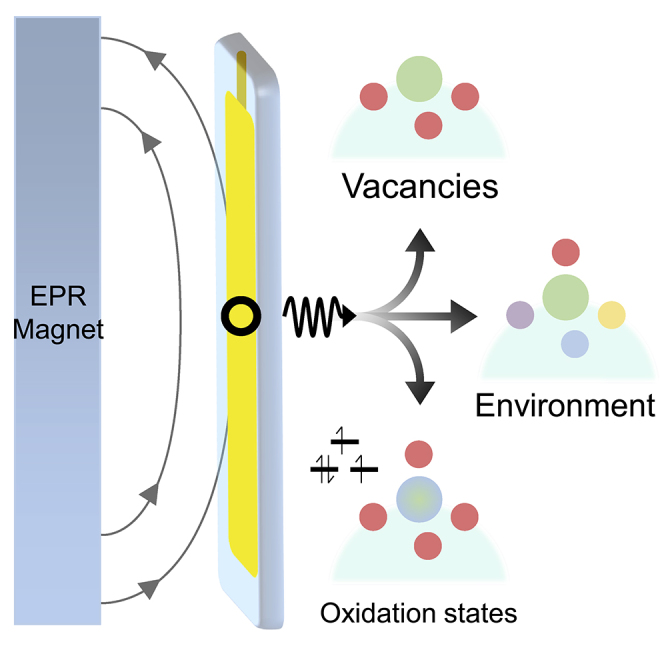

## Introduction

Energy storage is a key aspect in the implementation of green energy as it allows for carbon-neutral electricity to be kept and utilized at will. Electrochemical processes figure prominently in the energy storage solutions, such as artificial fuels and batteries. Toward the development of new materials and devices, spectroscopy plays an important role as it allows for phenomena to be correlated with specific physical events. One such technique is electron paramagnetic resonance (EPR) spectroscopy, which is used to study unpaired electron spins. EPR has so far been used in studying *operando* electrolyte phenomena in redox flow batteries^1^, ^2^, ^3^ or fuel cell membranes,^4^^,^^5^ but its utility in studying conductive solid-state materials such as electrocatalysts remains limited.

EPR can be challenging to perform for electrochemical studies in the presence of conductors and high-dielectric electrolytes such as water, which both can affect the quality of the spectroscopic signal. Although these contradictions can prove debilitating to data collection, demand for new spectroscopic understandings of catalytic systems has fueled interest in how such challenges can be overcome. In this review, we provide an overview of EPR, the potential benefits of using EPR for characterizing electrocatalysts, how challenges in carrying out solid-state electrochemical EPR have been successfully overcome, and some of the recent insights that EPR has provided toward electrocatalytic materials in several key systems.

## Overview of solid-state electron paramagnetic resonance spectroscopy

**Figure 1:**
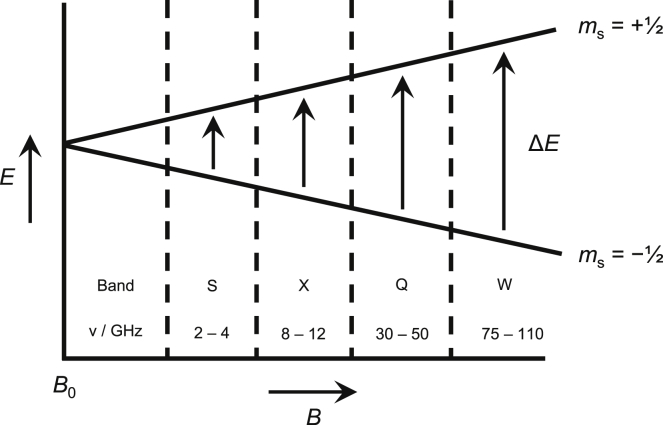
The Zeeman effect As the strength of a magnetic field is increased, the energy required to transition between spin states is also increased. The listed band frequencies are the operating frequencies under which EPR at each band is usually undertaken.

EPR spectroscopy is used to study the nature of unpaired (paramagnetic) electrons in a given system, based on transitions between their spin states. Since electrons are charged particles, their natural spins (often denoted as +1/2 (high spin) and –½ (low spin)) generate magnetic fields. Electrons can transition between spin states with the application of energy. Furthermore, the interaction of an electron’s magnetic field with an external magnetic field can change the energy required to induce a spin transition, a principle known as the Zeeman Effect (Figure 1), named after Pieter Zeeman who first observed that the absorption spectrum of a sodium flame splits in the presence of a magnetic field. EPR spectroscopy enables the user to measure the energy required to induce spin transitions under a given magnetic field strength, with the required energy being characteristic of a specific paramagnetic species.

EPR is often compared to its more well-known cousin, nuclear magnetic resonance (NMR) spectroscopy, which is focused on resolving nuclear spins. The relative sensitivities of the two techniques can be compared using the Larmor equation:

$$v=\left(\frac{\gamma }{2\pi }\right)B_{0}$$

Where ν is the frequency of the photon released (or absorbed), γ is the native gyromagnetic ratio of the particle of interest, and B_0_ is the magnetic field. Since an atom’s gyromagnetic ratio is directly proportional to the energy of the released (or absorbed) photon, and γ for an electron is three orders of magnitude greater than that of most nuclei, EPR is much more sensitive than NMR. This sensitivity makes EPR ideal for studying species at low concentrations such as in surface studies,^6^ which would include solid-state electrocatalysis.

As with NMR, the Larmor equation also points to increased magnetic field strength as another source of improved spectral resolution and sensitivity. However, unlike the gyromagnetic ratio, which is an innate property of a particle, magnetic field strength can be achieved with increasingly advanced infrastructure. As shown in Figure 1, the four most commonly used bands of microwave radiation for EPR are the S, X, Q, and W bands, with typical working frequencies ranging from 2 GHz (S-band) up to 110 GHz (W-band). Most published EPR studies are carried out using standard X-band spectrometers.

In practice, EPR spectrometers measure emitted microwaves as the applied magnetic field is varied. By measuring the magnetic field at which spin transitions take place, EPR can be used to calculate the *g*-factor, a unitless value that is characteristic of the paramagnetic oxidation state. For example, free electrons have an ideal isotropic g-factor of 2.0023.^7^ However, the EPR spectrum of any paramagnetic species undergoes variation due to the interaction of the electron with the surrounding environment. The primary source of variation is the nuclei with which the paramagnetic electron is associated with, allowing EPR to be used for the specific identification of oxidation states. In solid-state systems, where rotational degrees of freedom are finite, EPR spectra are further split into different g tensors, which are often described by a 3 × 3 matrix representing a three-dimensional coordinate system. The tensors arise as a result of interactions between the orientations of electron spins with the physically constrained solid crystal field as well as the applied magnetic field. Sometimes the EPR spectra of electrocatalysts in the solid state remain isotropic, often when the environment of the paramagnetic species retains a highly symmetric crystal field (i.e., tetrahedral, octahedral species with highly equivalent ligands). However, if the crystal field is elongated in one direction (i.e., axial), the spectra will split into perpendicular (g_⊥_) and parallel tensors (g_װ_). Finally, if the tensors have low symmetry, the spectrum will split into three separate tensors (i.e., rhombic) denoted by their coordinate direction (g_x_, g_y_, g_z_). The separation distance of the tensors in the EPR spectrum gives rise to the symmetry factor, which describes the overall symmetry of the paramagnetic center. For reference, a list of *g-*factors in electrocatalytic systems discussed in this review is provided in Table 1.

**Table 1:** Common g-factors found in electrocatalysts as discussed in this review

Species	Effective g-factor	g_*⊥*_	g_װ_	g_rhombic_	Reference
*Free electron*	2.0023				^7^
*Mo*^*3+*^	2.014				^8^^,^^9^
*Mo*^*5+*^*= O*		1.92	1.86		^10^
*Mo*^5+^*/S-coordinated*				1.97, 2.01, 2.05	^11^
*Co*^*4+*^		2.33	2.06		^12^^,^^13^
*Co*^*2+*^	∼5 (broad)				^12^^,^^13^
*Mn*^*4+*^*(low spin)*	1.83				^14^
*Ni*^*3+*^	2.172				^15^
*Cu*^*2+*^	2.14				^16^

The next important feature of EPR spectra is their line widths, which originate from the relaxation times of the electron spins. Relaxation time refers to the time that it takes for electron spins to reach equilibrium upon entering a state of disequilibrium. There are two types of relaxation time in EPR: spin-lattice or longitudinal relaxation (T_1_), and spin-spin or transverse relaxation (T_2_). T_1_ refers to the time that electron spins take to relax from a higher energy spin state back to the ground state upon removal of an applied magnetic field. While in the magnetic field, spins are aligned parallel to the magnetic field (or z direction), but upon removal of the magnetic field, these spins gradually return to a lower energy spin state, with the excess energy dissipating through the surrounding lattice structure. T_2_ originates from exchanges between nearby electrons with opposite spins; these spins can exchange their spins with each other. The time that it takes for these spins to exchange constitutes T_2_. These spins are not aligned with the applied magnetic field and hence are referred to as transverse as they exist within the transverse plane of the magnetic field (the x/y directions). For both types of relaxations, shorter relaxation times translate into broader linewidths due to the increase in uncertainty of the electron spin state. Solid-state EPR spectra are significantly broader than liquid-state spectra due to the increased proximity and interactions with neighboring spins; as a result, T_2_ is much shorter, resulting in linewidth broadening.

Electrons can also interact with spin-active nuclei, resulting in hyperfine coupling of a spectrum. The magnitude of a hyperfine interaction is quantifiable; furthermore, since each tensor has a vector component, different magnitudes of interaction can be applied to each tensor. The ultimate result is that, if the hyperfine interactions are large enough, the tensor of interest will undergo broadening and ultimately splitting. Dipole-dipole interactions between different unpaired electrons can result in another type of splitting known as zero-field splitting. Zero-field splitting takes place as a result of the presence of multiple, interacting unpaired electrons around a single nucleus, resulting in the existence of an additional, field-independent energy level that can be represented in a Zeeman diagram. Spins can transition between this field-independent energy level as well as the field-dependent energy levels, resulting in new splittings in an EPR spectrum. However, zero-field splitting is often extremely broad and difficult to detect, except at low temperatures. Furthermore, in the examples discussed here, there have been no demonstrated examples of zero-field splitting as the spectra arising from paramagnetic species have been more detectable and useful to electrocatalyst studies thus far.

Given these complications, a common misconception about g-factors is that they are constant. Indeed, our inclusion of Table 1 seems to encourage this belief. However, although the value of g-factors is heavily dominated by the associated paramagnetic oxidation state that they are associated with, the presence of the variables discussed above means that significant variation can take place. The primary example is how tensor splitting results in an ordinarily isotropic g-factor splitting into multi-dimensional tensors, but even linewidth broadening or the presence of multiple paramagnetic species can appear to vary the positions of collected EPR data. To assist in the interpretation and modeling of potentially complex data, open source tools such as EasySpin (a MATLAB toolbox) and SimLabel (a user-friendly interface version of EasySpin for non-MATLAB users),^17^ can be quickly learned even by inexperienced users to model output signals with ease.^18^ Realistically, given the challenges of interpreting EPR data, simulations are the ideal benchmark for analyzing and comparing EPR data. Finally, since this review is directed toward non-users, we recommend the introductory text by Chechik et al.^19^

## Experimental setups for studying electrocatalysts by electron paramagnetic resonance spectroscopy

**Figure 2:**
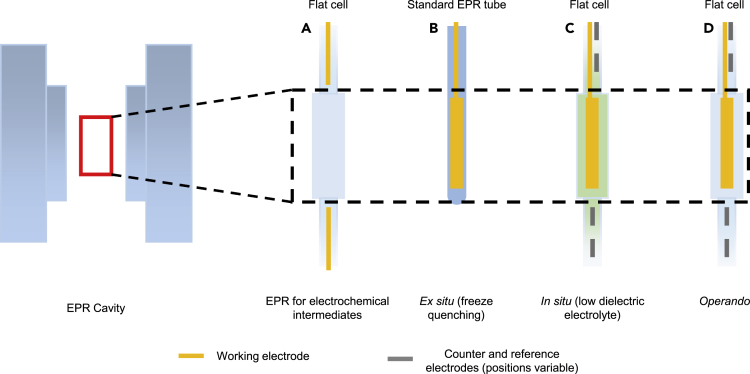
EPR cell configurations used for electrochemical studies described in this review

Paramagnetic species are central to catalysis due to the outsized role that unpaired electrons have in determining chemical reactivity.^20^ Since electrochemistry often involves the direct generation of charged species by sequential electron transfer, EPR and electrocatalysis can be quite complimentary. As with any liquid-based EPR system, practical applications are complicated by the absorption of microwaves by high-dielectric electrolytes. Electrolytes by definition have high-dielectric constants; traditionally, this issue could be overcome by the use of flat cells to minimize the cross-section of the electrolyte, minimizing dielectric loss.^21^, ^22^, ^23^ Electrochemical intermediates could then be studied by placing two electrodes on opposite ends of the cell, outside the cavity, such that generated species diffuse through the cavity during analysis. This setup is often used to study liquid-phase paramagnetic electrochemical intermediates (Figure 2A).

In order to study a solid electrocatalyst material by EPR, however, the electrode itself must be placed within the cavity in the direct path of the magnetic field. As conductors, electrode materials reflect electromagnetic radiation including microwaves. In effect, a shielded sample cannot be locked onto by the spectrometer, and spectra cannot be obtained. Conductive EPR is only possible if the amount of sample in the spectrometer is minimized such that the shielding is not overwhelming. Hence the earliest studies of conductive, paramagnetic metals were performed by dispersing microparticles in a low dielectric medium,^24^ or reducing the cross-section of the sample in the cavity.^25^^,^^26^ In electrochemical settings, the combination of both spatially constrained electrodes and electrolytes further creates diffusion limitations that vastly increase the resistance of the cell, requiring the user to use both a good potentiostat and carefully mind cell design. There are also reports of placing wire electrodes within the cavity to study reactions of soluble intermediates at or between electrodes.^27^^,^^28^ The success of these studies arises from the use of thin wire electrodes, which have a minimal cross-section, but for studying solid-state catalysts, the lack of material presence in the cavity would ultimately minimize already weak signal.

Consequently, *ex situ* measurements, where a reaction is quenched before analysis, have utility for EPR assuming the active state can be successfully trapped (Figure 2B). However, *ex situ* electrochemical EPR studies of aqueous-based catalysts where freeze-quenching is utilized, benefit from the fact that ice does not absorb microwaves, greatly reducing electrolyte dielectric loss. As a result, cell design is less stringent and standard quartz EPR tubes can be used for analysis. Furthermore, EPR is more sensitive at lower temperatures due to the longer relaxation time of paramagnetic species. On the other hand, *in situ* measurements can be carried out under experimental applied potentials in non-operational electrolytes (such as organic solvents) particularly in reactions where water (a high-dielectric electrolyte) is involved (Figure 2C).^29^ Organic electrolytes have recently demonstrated great value in studying aqueous reactions specifically as they do not interact with catalyst materials under operational potentials, making it possible to study electrocatalysts in only the presence of an applied potenital.^8^^,^^30^^,^^31^ The primary downside of organic electrolytes is their poorer conductivities, making it difficult to apply current without overloading the potentiostat. To circumvent higher electrolyte resistance, higher concentrations of the electrolyte can be used, and the reference electrode should be positioned as close to the working electrode as possible without being in the cavity. Finally, *operando* measurements are in theory the most ideal measurements as they capture a system in its natural state (Figure 2D). However, they also tend to be the most challenging for aqueous-based reactions for the reasons described above. Fortunately, the high conductivity of aqueous electrolytes often means that the electrochemical setup is often easier than for *in situ* measurements. As a summary, the general characteristics of each setup are listed in Table 2.

**Table 2:** Comparison of different setups of EPR

Setup	Ex situ	*In situ*	Operando
Sensitivity	Highest	Moderate	Lowest
Captures catalyst behavior?	Variable	Partial	Full
Ease of setup (electrochemical)	Easiest	Hardest	Moderate

The two most important parts of any design are the cell that will go inside the cavity and the working electrode that will go inside the cell. To maximize signal while minimizing dielectric loss, the electrode must have as large an area as possible while being as thin as possible. For example, flat metal wires or thin films sputtered on flat EPR-silent substrates can both be effective. The flat section of the electrode must then fit inside the cell, which itself will go within the cavity of the instrument. The other parts of the electrode should be sheathed with inert material, i.e. glass, Teflon, solvent-resistant epoxy, to prevent interactions between the electrode and electrolyte.

For the cell, the most important component is the section that fits within the spectrometer. *In situ* and *operando* measurements are best carried out in flat cells but may not be needed depending on the electrolyte. The goal of the flat cell is, like with the flattened wire, to reduce the cross-section of the electrolyte in the EPR cavity. Additional ports attached to the flat cell are also needed to interface counter and reference (if needed) electrodes, but since these will be outside the cavity, their specific design is less stringent as long as they follow basic electrochemical principles, i.e. allowing for relative proximity to the working electrode. For *ex situ* aqueous measurements, cell design is less relevant as ice has a low microwave absorption profile.

**Figure 3:**
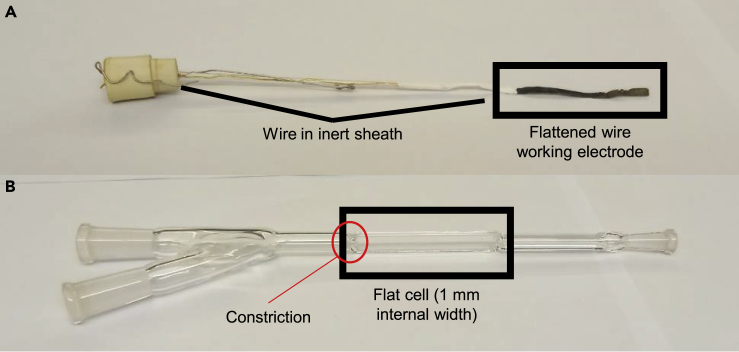
Cell design considerations for *operando* and *in situ* EPR The two most important components, as they fit within the cavity, are marked and labeled with a black rectangle. (A) The working electrode for EPR measurements, here consisting of a gold wire flattened on one end to fit within a 30 mm quartz flat cell. (B) Wilmad-LabGlass quartz electrochemical EPR cell used for cavity experiments in conjunction with a flat wire.

Since flat cells are generally the most difficult part to home-make, oftentimes it is most convenient to acquire one commercially from EPR cell vendors such as Wilmad Labglass (Figure 3). The common electrochemical cell, which is designed for liquid-phase measurements, can be easily used for solid-state measurements as well. However, the commercial version has a constriction between the flat cell and the top of the port, which can hinder electrocatalytic experiments as diffusion from the electrode within the flat cell to the reference electrode is constricted. This constriction can be avoided in a custom cell to improve conductivity.

## Electron paramagnetic resonance spectroscopy compared to other techniques

Given these complications, it is important to emphasize why EPR is still valuable in providing information on solid-state electrocatalysts. As discussed above, the unique properties of EPR make it notably available, sensitive, and informative in the study of paramagnetic atoms (Table 3). Two other techniques that are often utilized to provide similar information are X-ray absorption spectroscopy (XAS) and X-ray photoelectron spectroscopy (XPS). Although both techniques can be used to study non-paramagnetic species, they also have higher limits of detection in the single-digit atomic percentage range. Both techniques also collect element-specific data in a sample and can therefore be limited in their abilities to study oxidation states of interest. EPR in contrast has ppm level sensitivity and is focused on paramagnetic oxidation states.

**Table 3:** EPR compared to other techniques

Technique	EPR	XPS	XAS
Sensitivity	ppm	%	%
Availability	Relatively common	*Ex situ* – common *In situ*/*Operando* – rare	Limited mostly to synchrotron facilites
Target atoms	Paramagnetic atoms or vacancies on atoms	All elements	All elements

However, while the relative sensitivity of EPR is impressive, it is ultimately the availability of EPR that greatly distinguishes it from the other two. XAS is typically limited to synchrotron facilities, and the time constraints in such facilities often means that specialized, surface-sensitive configurations are less accessible to common users. XPS is surface-sensitive, but using it for *in situ* or *operando* studies requires complicated equipment configurations to achieve ambient conditions.^32^ In contrast, in the combined experiences of the authors across institutions around the world, standard X-band EPR spectrometers are more accessible than competing techniques and faster to measure due to its lower cost and easier infrastructure requirements.

On a final note, EPR has the capability to study the coordination of paramagnetic atoms that other techniques would find difficult, such as vacancies or protons. Vacancies in particular have notable EPR spectra since the loss of a coordinating atom automatically leaves a free electron on a paramagnetic center—something that not all techniques are effective at.^33^ Considering that vacancies are where reactants are often coordinated to catalytic centers, EPR has a unique role in providing basic catalyst characterization.

## Electron paramagnetic resonance spectroscopy studies of battery and supercapacitor electrodes

EPR techniques have proven successful in studying battery systems via both *in situ* and *operando* setups,^34^ which is unsurprising given that the paramagnetic metals that serve as anodes have long been studied by EPR.^24^^,^^35^, ^36^, ^37^, ^38^, ^39^, ^40^ Since EPR can be used to study the coordination environment of paramagnetic atoms, it can be used as a measure of the porosity of alkali metal deposits in batteries that contribute to battery degradation^35^, ^36^, ^37^ or how Li interacts with electrode materials.^39^^,^^40^ Transition metal oxidation states common in electrode materials are also good targets for EPR, and paramagnetic species have been reported for Ru-,^41^ V-,^42^^,^^43^ Co-,^44^^,^^45^ Mo-,^46^ Mn/Ni-,^47^, ^48^, ^49^, ^50^, ^51^ and Cu-containing^52^^,^^53^ electrodes. EPR can also be used to examine the dissolution of transition metal components of battery electrodes during operating conditions.^54^^,^^55^

The use of EPR for studying batteries has been more prolific than for electrocatalysts because the electrolyte is typically confined into a semi-solid thin film within the EPR cavity, minimizing dielectric loss. Similarly, studies of carbon-based supercapacitors minimize dielectric loss under *operando* conditions through the use of an electrode setup that fits into a 1 mm normal quartz EPR capillary.^56^, ^57^, ^58^ Supercapacitor studies are benefited as they mostly study free electrons; the broader nature of paramagnetic species in transition metal electrocatalysts requires greater surface area. Electrolyte confinement can be utilized for electrocatalysts through the use of membrane-based EPR cells,^4^^,^^59^ which have to this point mostly been used to study membrane behavior. It remains to be seen whether or not an *operando* fuel cell-style cell could be of use to study the catalysts themselves under *operando* conditions, or if the presence of decomposition products from the membrane would overwhelm potential signals. However, these cells have the advantage that catalysts can be studied under ultimate commercial conditions.

## Electron paramagnetic resonance spectroscopy for solid-state electrocatalyst characterization

**Figure 4:**
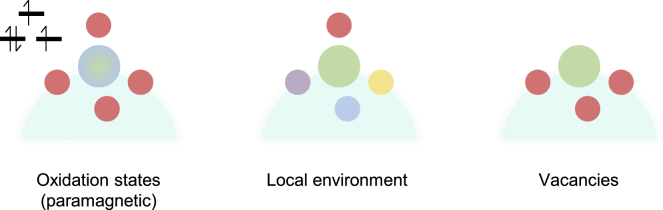
Conceptual diagram of three topics that EPR is excellent for studying in solid-state electrocatalysts

Solid-state spectroscopy of electrocatalytic materials is a relatively new field of study that has been necessitated by recent interest in electrochemical-based fuel systems, especially those reactions critical to fuel cells and electrolyzers. In the following section, we discuss a number of approaches that have been reported for the characterization of electrocatalytic systems via EPR with a focus on three general topics—the characterization of paramagnetic oxidation states, the local environment of a given paramagnetic oxidation state, and the quantification of vacancies (Figure 4). These three topics are ideal for EPR as they rely on the strengths of EPR in order to function properly. In the case of the first two topics, the species of interest must be paramagnetic, whereas vacancies by nature are paramagnetic. Although limiting, this drawback does not necessarily stop the useful collection of data by EPR.

## Examination of catalytic paramagnetic oxidation states – Co O_2_ evolution reaction catalysts

**Figure 5:**
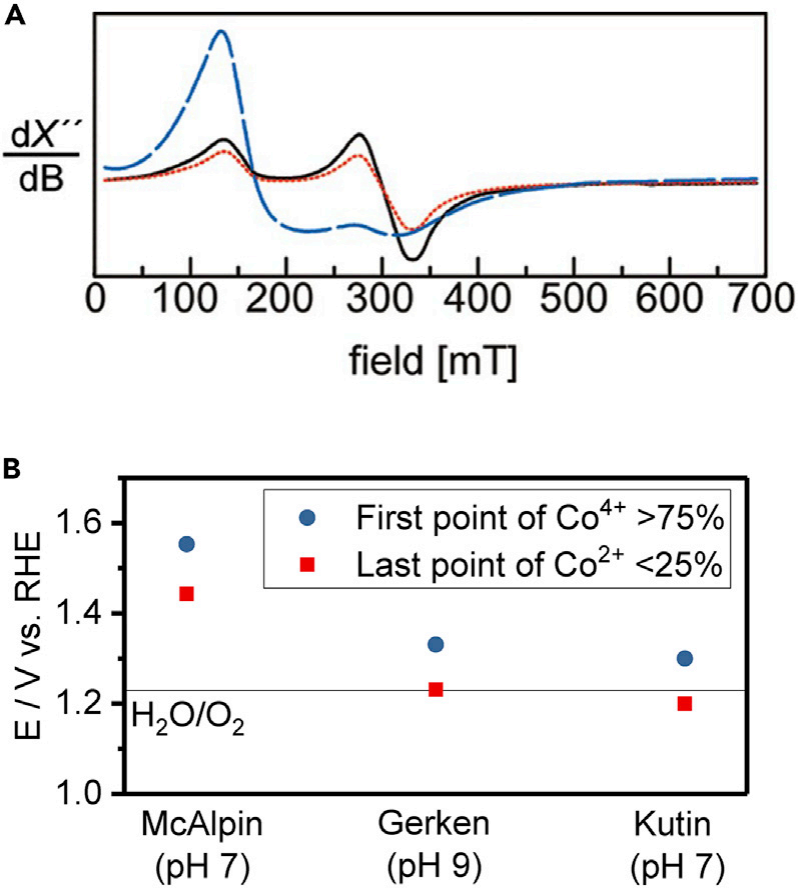
EPR characterization of CoP_i_ electrocatalysts for the OER (A) EPR spectra of CoP_i_ catalyst deposited at 1.03 V vs. NHE (blue curve), 1.14 V vs. NHE (red curve), and 1.34 V (black curve) vs. NHE. Reprinted with permission from McAlpin et al.^12^ Copyright 2010 American Chemical Society. (B) Comparison of the reported transition point between Co^2+^ and Co^4+^ in three studies discussed in this review. The thermodynamic OER potential is shown for comparison and all values (if needed) were corrected to RHE.

Co-containing electrodes are of interest to electrochemistry not only as battery cathodes but also as water oxidation catalysts. Amorphous electrodeposited Co oxide films deposited from phosphate (CoP_i_) and borate (CoB_i_) electrolytes display some of the highest reported activities for the O_2_ evolution reaction (OER) for non-noble metals.^60^^,^^61^ As these films lack any long-range structural order that could be the basis for activity, studies into the molecular origins for their catalytic activity are important not only for fundamental reasons but also for the design of new catalyst materials. Initial *ex situ* EPR studies of these films were aided by the assumed persistence of Co oxidation states during freeze-quenching, allowing thick films of prepared material to be manually delaminated and loaded into EPR tubes.^12^^,^^13^ In both studies, Co^2+^ species were found to disappear in favor of Co^4+^ prior to the onset of the OER. The recognized signal for high-spin Co^2+^ is a broad transition ∼ g = 5, whereas Co^4+^ exhibited a narrower transition ∼ g = 2.3 (Figure 5A), which was consistent with model compounds. No other species, for example, high-spin Co^3+^ (a paramagnetic configuration of an otherwise EPR-silent species) were detected. However, the Co^4+^ signal was severely attenuated with increasingly anodic potentials,^13^ which often takes place when paramagnetic centers become too concentrated in a sample, due to excessive hyperfine interactions.^62^ This attenuation seems exclusive to Co^4+^ in the Co system, as no attenuation was found when the catalyst was dominantly (if not almost entirely) Co^2+^.

Although catalyst delamination was used, the same behavior prevails on the catalyst that is directly quenched under operating potentials.^63^ In this subsequent study, Co^4+^ formation was found to be roughly coincidental with the onset of the OER (+/− 100 mV). Across all three studies, the same trend emerges—formation of Co^4+^ from Co^2+^ takes place around, or slightly beyond the onset of the OER. This trend is especially apparent once the results are normalized for pH (Figure 5B). It is likely then that Co^4+^ is the resting state of the catalyst, which then proceeds with O_2_ evolution once the thermodynamic potential is reached. In all three cases, it is apparent that Co^4+^ formation at the expense of Co^2+^ is the most significant electrochemical event prior to OER activity. Most significantly for experimental design, the catalyst can be analyzed on a conductive electrode without the need for the destruction of the original sample, greatly facilitating future electrochemical measurements.

An opposite upfield shift was reported in a mixed Co-Cu oxide system.^16^ To acquire *operando* spectra, this study was performed using a carbon sheet with dispersed catalyst as the working electrode. Unlike the amorphous Co oxide films, the CoCu oxides are nanoparticles with a bulk rock-salt structure, meaning that it is antiferromagnetic and as a result, EPR silent (except for uncoordinated surface sites), possibly also explaining the high background of the EPR spectra.^64^ These surface states disappeared with longer exposures to 0.1 M KOH, possibly due to coordination arising from hydroxide groups according to the Pourbaix diagram of Co.^65^ Assessing the nature of the new species is difficult without more information, but the final spectrum acquired during the OER seems to suggest that a change in catalyst structure as a result of Cu dissolution, which was also demonstrated by EPR. This study, therefore, highlights how EPR is useful in tracing the ability of dopants to change the behavior of new catalysts compared to parent systems.

## Examination of mixed metal catalysts – Mn-Ni O_2_ evolution reaction catalysts

Like Co, pure Mn oxides show promise for OER catalysis, albeit at lower activities. Mn is the OER catalyst of nature, found as part of the Mn_4_Ca complex of Photosystem II. EPR has been an important tool for understanding this biologically important system. For example, the discovery of a characteristic ≥18 hyperfine line spectrum was attributed to a Mn_4_Ca cluster with Mn nuclei in the 3 + or 4 + oxidation states.^66^ Paramagnetic Mn species with specific oxidation states (2+, 4+) display characteristic EPR spectra due to the nuclear spin of Mn (+5/2), resulting in spectra with significant hyperfine coupling for both Mn^2+^ and Mn^4+^.^67^, ^68^, ^69^ Similarly to Photosystem II, OER-active Mn-based catalysts contain Mn^3+^ and Mn^4+^,^70^ and much work has gone into exploring the unique EPR spectra characteristic of Mn^3+/4+^-based clusters both in natural and artificial systems.^71^, ^72^, ^73^ Mn oxides stand out from first-row transition metal oxide OER catalysts for their better stabilities in neutral and acidic conditions, even if they are less active.^74^^,^^75^ These traits (better stability, wide oxidation state range, lower activity) suggest that Mn atoms could better be used as a source of oxidation state control in other, oxidation-state dependent catalysts.

**Figure 6:**
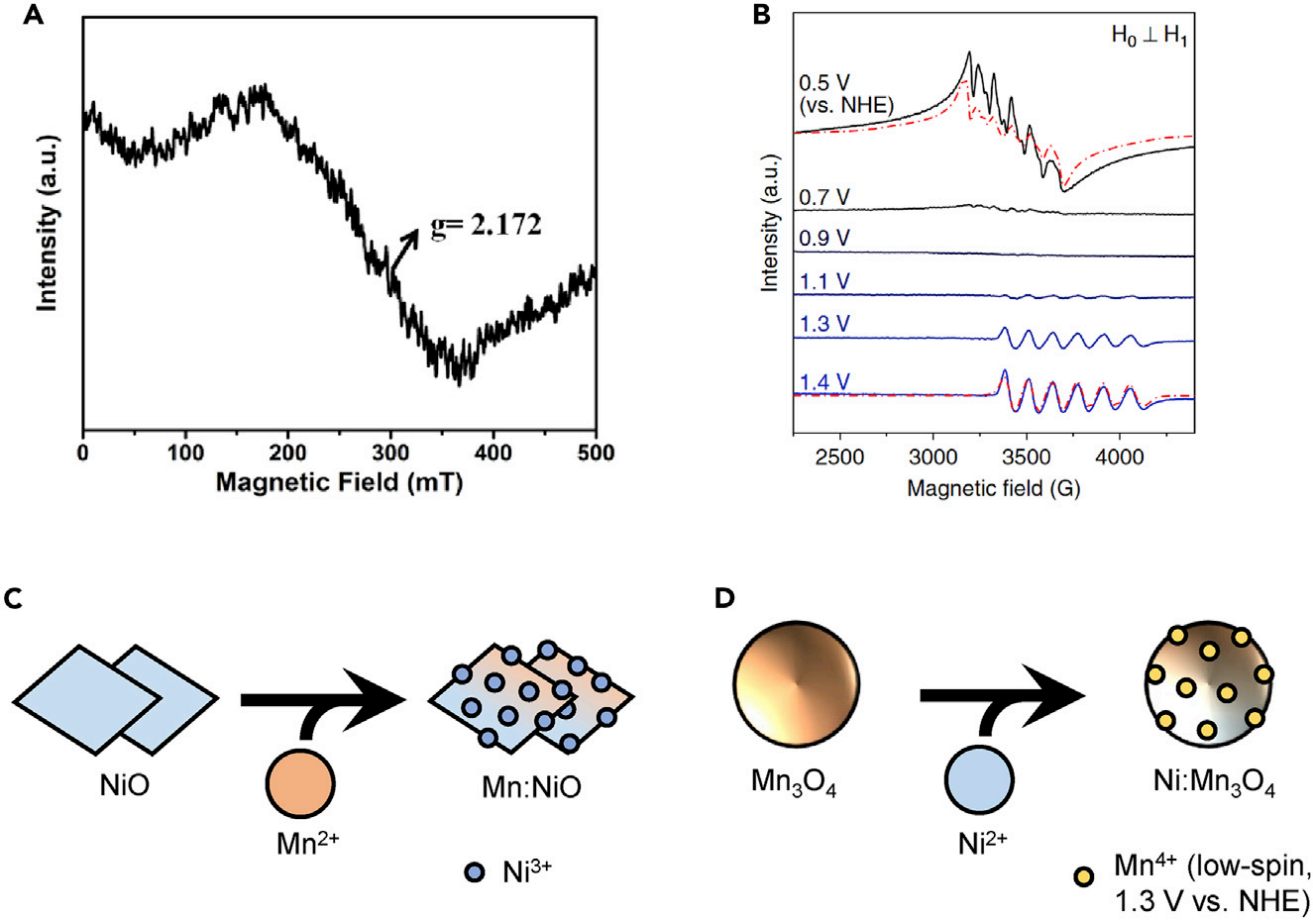
Mn-Ni mixed oxides for the OER studied by EPR (A) X-band EPR spectrum of ground state Mn-doped NiO nanosheets, with the *g*-factor of Ni^3+^ labeled. Reprinted with permission from Tian et al.^15^ Copyright 2018 American Chemical Society. (B) X-band EPR spectra of the emergence of low-spin Mn^4+^ species under OER potentials from an electrode of Ni-doped Mn_3_O_4_ nanoparticles. Mn^2+^ spectrum is visible in the top row; starting at 1.1 V vs. NHE, the low-spin Mn^4+^ species is observed. Modeled data, generated using EasySpin, is provided as the dotted red spectra. Reprinted with permission from Park et al.^14^ Copyright 2020 Springer Nature. (C and D) Depictions of how the dopant atom added to the base material in both cases yields EPR-visible paramagnetic species Ni^3+^ and low-spin Mn^4+^.

Since unique paramagnetic centers have unique *g*-factors, EPR shows promise for studying the presence of multiple oxidation states within a given catalyst. Oftentimes, the mixing of transition metals in a catalyst will lead to the synergy between neighboring metal centers, allowing for the novel activity of the mixed catalyst. For example, the incorporation of Mn in nominally NiO oxide nanosheets (rock-salt structure) can be used to stabilize Ni^3+^ at ground state, resulting in superior OER activities compared to pure Mn_2_O_3_ or NiO.^15^ Mn^2+^ was not observed in the EPR spectrum, suggesting that Mn was mostly present as Mn^3+^ (Figures 6A and 6C). Ordinarily, Ni^3+^ formation from Ni^2+^ takes place during a one electron transfer prior to the OER as a result of the Ni^2+/3+^ redox couple taking place prior to the thermodynamic OER potential,^76^ possibly contributing to sluggish OER activity of pure Ni oxides based on their large Tafel slopes compared to other transition metal oxides.^77^ The stabilization of Ni^3+^ prior to the OER possibly explains the activity of Mn-doped NiO as Ni oxidation step is not necessary during anodic polarization.

Alternatively, Ni doping into spinel Mn_3_O_4_ nanoparticles also can result in a more active Mn-Ni oxide OER catalyst.^14^ By tracing the behavior of the catalyst on polarization using *ex situ* freeze-quenching, it was observed that a new, low-spin Mn^4+^ species was found to emerge, leading the authors to conclude that Ni stabilized low-spin Mn^4+^ in Mn_3_O_4_ within a compressed crystal field (Figures 6B and 6D). Notably, at no time were any EPR spectra for Ni^3+^ observed, demonstrating that the oxidation state of Ni remained Ni^2+^. Although the catalyst electrode was composed of the doped nanoparticles on a NiO support, OER activity was nonetheless greater than the support alone. Therefore, it is reasonable to argue that the new low-spin state of Mn is responsible for conferring OER activity, and not the presence of dopant Ni atoms.

## Local environments by electron paramagnetic resonance spectroscopy – Mo-based systems for the H_2_ Evolution Reaction (HER)

In our own experiences, EPR has proven helpful in analyzing less-stable oxidation states of Mo that can be stabilized or trapped under various conditions as well as their local environments. Mo is a d^6^ element with multiple oxidation states under ambient conditions, and Mo-based catalysts are some of the most-active noble metal-free catalysts for the HER.^78^, ^79^, ^80^ Existing non-EPR studies of Mo-containing catalysts during the HER generally agree that a reduced state of Mo is responsible (i.e., <Mo^6+^),^81^^,^^82^ but the variety of possibly involved oxidation states makes it difficult to pinpoint a specific charge. Theoretical mechanistic studies suggest that multiple Mo oxidation states participate in the HER,^83^ but our interest has been in identifying the active species, most likely a hydride- or H∗-containing species, that resting state of the catalyst. The earliest *ex situ* EPR study of amorphous Mo sulfide (*a*-MoS_x_) utilized Mo sulfide nanoparticles reduced by sodium dithionite in lieu of cathodic potentials to suggest the presence of Mo^5+^ (which became more intense after reduction).^10^ Since no electrodes were involved in the EPR part of the study, this system allowed for the quick isolation and study of Mo sulfides without the need for a hybrid EPR/electrochemical setup.

**Figure 7:**
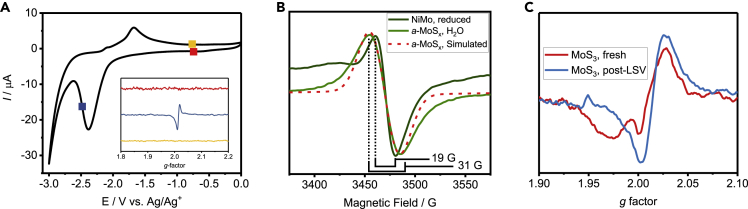
EPR for characterization of Mo during the HER (A) Cyclic voltammograms of NiMo in 0.2 M Bu_4_N PF_6_/THF before (black) and after (green) electrolysis in 0.1 M KOH. *Inset*, EPR spectra of Mo^3+^ signal before (red), during (blue) and after (yellow) the NiMo electrode was held at −2.5 V vs. Ag/Ag^+^. Reprinted with permission from Bau et al.^8^ Copyright 2020 American Chemical Society. (B) EPR spectrum of trapped Mo^3+^ hydride in *a*-MoS_x_ (light green). The spectrum of Mo^3+^ formed from NiMo under organic cycling in (a) is shown for comparison (blue trace in (a), dark green). A simulated spectrum for *a-*MoS_x_ is provided in red. (C) EPR spectra of *a*-MoS_x_ deposited with a cathodic final potential (blue, similar to 6b) and an anodic potential (red) with annotated characteristic *g*-factors corresponding to Mo^5+^ signals. Figures (b) and (c) reprinted with permission from Bau et al.^85^ Copyright 2022 Springer Nature; (c) was compiled and re-annotated with data from both references.

In our initial study of Mo in NiMo alloys, Mo^3+^ was found to be present during *in situ* (cathodic potential) EPR in organic electrolyte (Figure 7A). Lacking a reducing agent or reasonable means that could be used for *ex situ* studies, the catalyst electrode was electrochemically reduced in organic electrolyte. *In situ* conditions were necessary due to the instability of the Mo^3+^ state; the catalyst would re-oxidize upon the loss of applied potential. Furthermore, the use of THF as organic electrolyte lowered the microwave absorption profile for better signal resolution. The kinetics of the underlying reaction could also be slowed to the point that specific oxidation states could be electrochemically resolved.^30^ To demonstrate the connection between aqueous and organic systems, cyclic voltammograms of the catalyst in organic electrolyte with increasing amounts of water were used to show how the onset of the HER related to reduction events. Meanwhile, attempts to capture the same species by XAS,^84^ or by *ex situ* or *operando* EPR proved unable to resolve the species of interest. The EPR spectrum of Mo^3+^ also provided insight into its local environment due to the presence of axial symmetry, demonstrating that it had a distorted crystal field. Quantitative EPR was therefore used to compare the number of spins to the total amount of Mo (acquired using inductively coupled plasma measurements), finding that only a minority of the Mo species—likely only a surface layer—formed Mo^3+^. Therefore, the anisotropy implies that Mo^3+^ is surface bound, also highlighting the remarkable sensitivity of EPR.

We later used a similar approach to re-examine *a*-MoS_x_.^85^ While *in situ* electrochemical measurements could (and were) used, the Mo^3+^ signal could also be trapped *ex situ* by ending the electrochemical deposition (by cycling) protocol for *a*-MoS_x_ suggested by Merki et al.^86^ at H_2_-evolving potentials. The resulting catalyst exhibited a Mo^3+^ signal that was both broadened and isotropic compared to that found in NiMo (Figure 7B). The isotropy suggests that active sites are present throughout the entirety of the porous catalyst and oriented randomly. However, it was the broadening (31 G for *a*-MoS_x_ vs. 15-19 G for NiMo or *a*-MoS_x_ reduced in organic, aprotic electrolyte) that proved interesting as it illustrated the presence of hyperfine coupling from a neighboring atom. As the most terrestrially abundant form of S is not spin-active, the only possible remaining candidate was H, which could be confirmed by EasySpin simulations. By other means, the presence of a metal hydride was confirmed. EPR was able to provide direct experimental evidence for a Mo^3+^-H active site in *a*-MoS_x_. EPR has also demonstrated that both *a*-MoS_x_ and NiMo behave similarly in electrochemical conditions, suggesting that a common mechanism between Mo catalysts exists for the HER.

Although differences in spectra exist between *ex situ* reduced Mo sulfide nanoparticles and electrochemically reduced Mo electrocatalysts, the results are not necessarily contradictory as Mo^5+^ was found in *a*-MoS_x_ when the deposition was ended on the anodic edge (Figure 7C). The electrode-based system that we utilized allowed us to capture both species, allowing for the elucidation of a complete process.

## Electron paramagnetic resonance spectroscopy for the examination of the roles of vacancies in electrocatalysis – NiFe O_2_ evolution reaction catalysts

Amongst the most well-studied multi-metallic electrocatalyst systems is Fe-containing Ni oxide, generally recognized as one of the most active earth-abundant OER catalysts.^87^, ^88^, ^89^ Although Ir- or Ru-based are still more active, the orders of magnitude abundance of Ni and Fe over noble metals makes them more realistic for practical implementation. The composition and amount of Fe dissolved in the Ni oxide structure is considered critical to high activity, and Fe is recognized as being important to carrying out the OER at low overpotential.^90^, ^91^, ^92^, ^93^ Like for Co oxides, amorphous NiFe oxides register high OER activities.^77^

**Figure 8:**
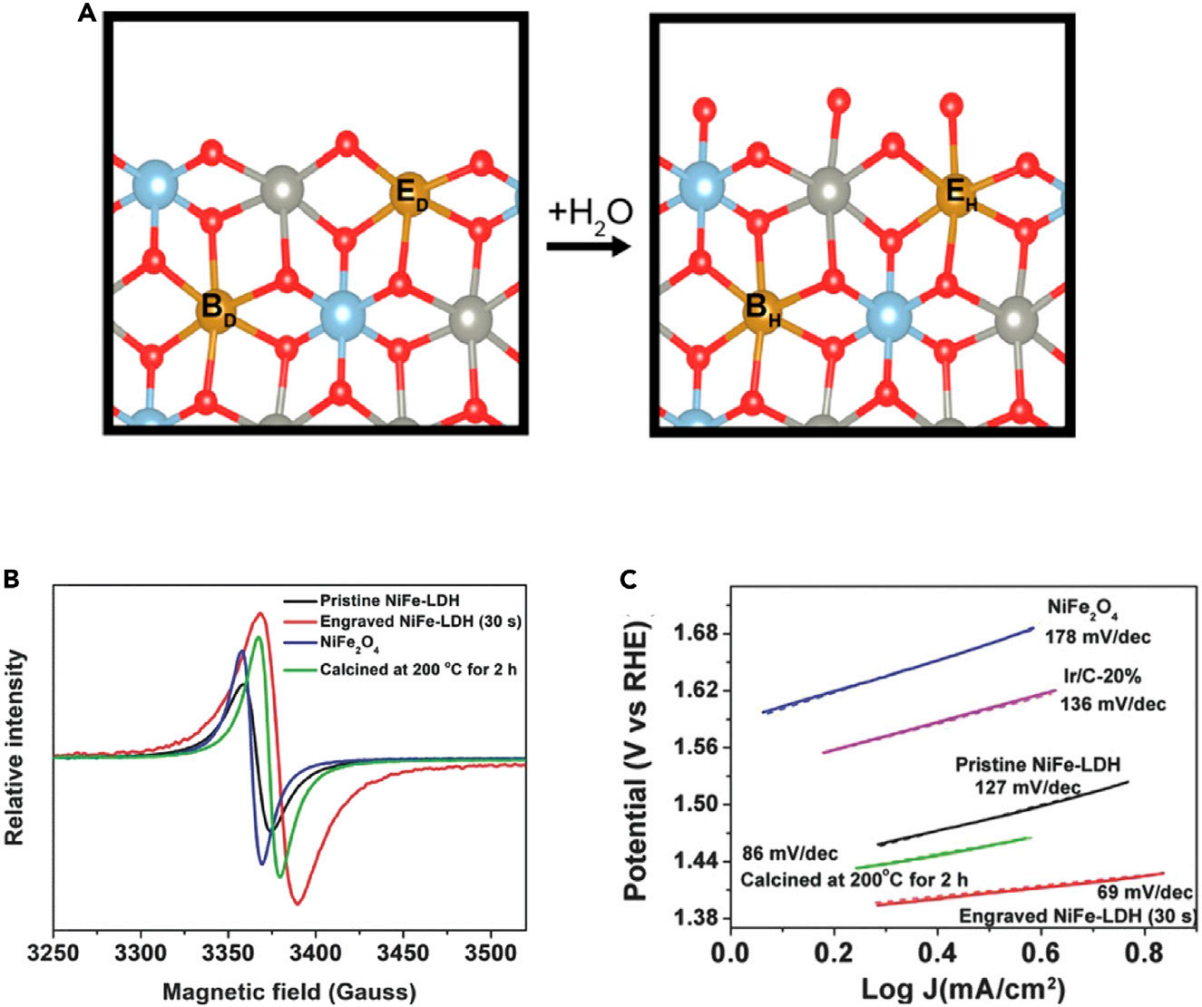
EPR studies of NiFe oxide (A) Dehydrated and hydrated forms of Ni/Fe LDH (orange) surrounded by Al (blue), Zn (gray) and O (red) atoms. E, edge sites; B, bulk sites. Reprinted with permission from Sayler et al.^98^ Copyright 2020 American Chemical Society. (B) EPR spectra of NiFe LDHs as prepared (black), after reducing flame treatment (red), and calcined at 200°C for 2 h (green). NiFe_2_O_4_ powder is studied as a control (blue). (C) Tafel plots of the same catalysts for the OER reaction in 0.1 M KOH. Color scheme is the same as (b), with the exception of Ir/C (purple) which was added for comparison. Reprinted with permission from Zhou et al.^94^ Copyright 2018 American Chemical Society.

The oxidation states of NiFe oxides during the OER are potentially plentiful,^30^^,^^90^ meaning that a good experimental setup would be needed for proper EPR studies. However, like Co^4+^, NiFe systems suffer from internal magnetic interference arising from local interactions at high concentrations, making *in situ* and *operando* mechanistic studies with EPR difficult. Fortunately, EPR has been useful in exposing another aspect of NiFe oxide function—the presence and role of vacancies.^94^, ^95^, ^96^, ^97^ The vacancies seem to arise from the facile nature by which water associates and dissociates with surface Ni and Fe centers, as demonstrated by Sayler et al. on layered double hydroxides of Zn and Al doped with Ni and Fe atoms at low concentrations (Figure 8A).^98^ Both of Ni- and Fe-doped ZnAl LDH particles exhibit EPR peaks at the free electron value when dried under vacuum which disappears on rehydration, suggesting that these spectra arise from uncoordinated vacancies on the metal centers.

Furthermore, different groups have demonstrated that the size of the free electron peak arising from vacancies can be related to OER activity. For example, Zhou et al. found that NiFe catalysts of varying activities can be prepared by carefully changing the heat treatment used post-preparation, postulating that each heat treatment introduces different amounts of oxygen vacancies into the structure (Figures 8B and 8C).^94^ The benefit of studying NiFe oxides with EPR is that no particular setups are needed, as long as the catalysts are consistently prepared, such as by pre-drying before measurement. The free electron is also not affected by the concentration of paramagnetic species, so EPR can be used to compare the presence of vacancies to OER activity even if other features of the sample are unmeasurable by EPR.

## The role of vacancies in electrochemical CO_2_ reduction on Cu metal-organic frameworks

CO_2_ electroreduction is a promising field of study for the recycling of waste CO_2_ into useful hydrocarbon products, but its study by EPR is further complicated (in addition to the other challenges discussed above) by poor CO_2_ solubility in water, and only *ex situ* setups in the absence of CO_2_ have so far been reported. Nonetheless, as with NiFe systems in the OER, EPR is useful in these providing insight into the role that vacancies play in CO_2_ reduction. For example, the amount of uncoordinated Cu^2+^ (as measured by the free electron peak) in the Cu-MOF HKUST-1 can be increased by thermal treatment, leading to improved Faradaic efficiency (FE) for ethylene production.^99^ By tracking the deterioration of free electrons, Kang et al. also demonstrated that the FE for formic acid production in another Cu-MOF was directly correlated with the amount of remaining uncoordinated Cu^2+^ sites.^100^ The loss of such sites was due to the reduction of Cu^2+^ to Cu^+^. Similarly, anodized Ti has the activity for electrochemical CO_2_ to methanol conversion dependent on the amount of Ti^3+^ and oxygen vacancies formed during Ti anodization.^101^ The authors had been unable to properly study these vacancies using XPS,^33^ underlining the value of EPR over other techniques for studying paramagnetic, uncoordinated materials. Based on these studies, it is likely that, much as how water coordinates to vacancies in NiFe to carry out the OER, vacancies in these materials serve as coordination sites for dissolved CO_2_.

## Outlook

In this review, we have sought to provide an overview of research seeking to utilize EPR to characterize the behavior of solid-state electrocatalysts with an eye to the experimental details that make such characterization possible. Given the role and range of transition metal oxidation states present in a variety of electrocatalysts, the local environments of these atoms, and the role of vacancies in catalyzing energy reactions of interest, EPR retains great potential as a spectroscopic tool. To complement some of these early discoveries, the development of several topics is important for future methodologies.

1. Substrate and electrode materials for the electrode of interest—liquid-phase EPR measurements are not a novel concept and indeed are well-established through the use of quartz flat cells; however, the combination of electrodes and liquids within the EPR cavity adds significant complications to acquiring spectra. Although EPR-silent electrode materials can minimize or completely eliminate shielding effects (i.e., doped semiconductors such as indium-doped tin oxide^102^), the fact that micron-thick metal foils can still give rise to the meaningful signal under *in situ* conditions suggests that the overall dimensions of the material are just as important. Furthermore, some doped semiconductors may not be adequately stable in the acidic or alkaline electrolytes used for electrochemical studies. Sputtered noble metal electrodes are more commonly found and can provide adequate conductivity with ∼50 nm films. An EPR-silent substrate material such as uncontaminated mica sputtered with metal on both sides could effectively be used in the same manner as a foil with minimal shielding losses.
2. New systems in which solid-state electrochemical EPR would have specific value—a major theme of the EPR studies so far reported is that the ideal catalysts to be studied have dilute active sites as samples with concentrated paramagnetic species can yield attenuated spectra, albeit depending on the species. Nonetheless, catalysts with dilute active sites—namely, single-site catalysts—have recently been of interest for their unique reactivities and high activities per weight loading. Many of these catalysts have transition metal centers that undergo changes in oxidation state under catalytic conditions. These characteristics make EPR a potentially unique technique for its characterization.

**Figure 9:**
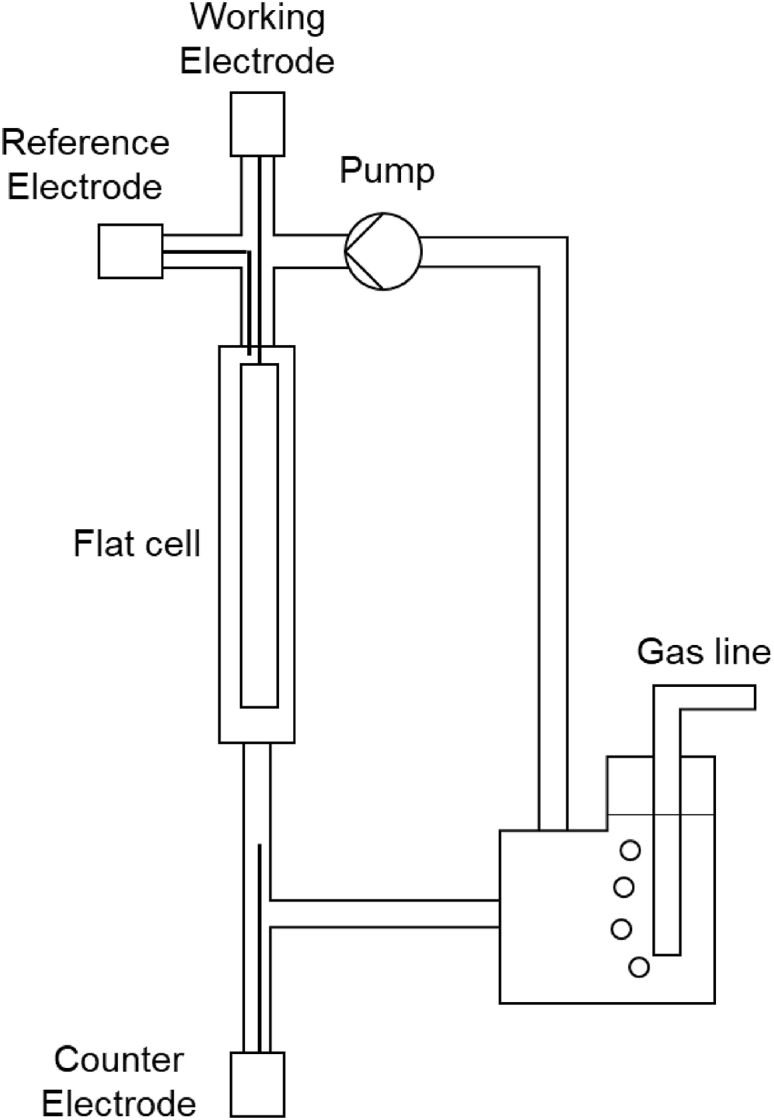
Diagram of a combined flow/electrochemical EPR cell for studying catalyst behavior for reactions where a gas is a reactant

Gas-phase reactions (especially CO_2_ and N_2_ reduction) have also lately been of interest to the electrochemical community and catalysts could be more thoroughly explored using EPR. Such gas-consuming reactions studied in the liquid state would require the combination of flow systems such as those utilized for fuel cell studies^4^ (to minimize diffusion limitations and allow for longer-term operation) with the electrochemical considerations discussed here. To allow for the study of such systems, a standard electrochemical cell could be modified to allow for separate pumping and degassing functions (Figure 9). By substituting quartz with EPR-silent polymers, the production and testing of such cells could be significantly sped up.^59^^,^^103^

Another group of reactions that have so far been unexplored using EPR is organic electrochemical reactions. Although aqueous reactions currently constitute an outsized portion of electrochemical studies due to the role of water as a proton and oxygen source in H_2_ and O_2_ evolution, fuel cell reactions, and CO_2_ reduction, organic electrochemical transformations have become a topic of interest. Given the lower dielectric constants of non-aqueous electrolytes, solid-state EPR could be valuable in demonstrating how and why different metal catalysts have different reactivities for different reactions. As an added bonus, EPR could simultaneously be used to study the mechanism of the reactions themselves.

EPR for solid-state electrochemical characterization is still in its infancy, but it is apparent that material selection and cell design are important to any future developments, especially for valuable *in situ* and *operando* studies. However, the minimal requirements described here are commonly found throughout the chemistry and materials science departments. We conclude that such applications might be readily applied in the future to provide unprecedented and highly available insights into the reactions that will be critical to the economies of the future.
